# Dietary phytochemical index and biomarker profiles are associated with colorectal cancer odds: integrating dietary intake with blood biomarkers

**DOI:** 10.3389/fnut.2026.1907770

**Published:** 2026-09-15

**Authors:** Juan Wu, Yubao Song, Meiyan Gao, Junwu Duan, Xuanxuan Ma, Dan Xue, Chengyan Zhang

**Affiliations:** 1 Department of Radiation Oncology, Shanxi Province Cancer Hospital / Shanxi Hospital Affiliated to Cancer Hospital, Chinese Academy of Medical Sciences / Cancer Hospital Affiliated to Shanxi Medical University, Taiyuan, China; 2 Department of Gastrointestinal Surgery, Shanxi Province Cancer Hospital / Shanxi Hospital Affiliated to Cancer Hospital, Chinese Academy of Medical Sciences / Cancer Hospital Affiliated to Shanxi Medical University, Taiyuan, China; 3 Department of General Internal Medicine, Shanxi Province Cancer Hospital / Shanxi Hospital Affiliated to Cancer Hospital, Chinese Academy of Medical Sciences / Cancer Hospital Affiliated to Shanxi Medical University, Taiyuan, China; 4 Department of Gastroenterology, Nanbu People's Hospital, Nanchong, China; 5 Department of Gastroenterology, Shanxi Province Cancer Hospital / Shanxi Hospital Affiliated to Cancer Hospital, Chinese Academy of Medical Sciences / Cancer Hospital Affiliated to Shanxi Medical University, Taiyuan, China

**Keywords:** colorectal cancer, gut permeability, inflammation, oxidative stress, phytochemical index, phytochemicals

## Abstract

**Background:**

Colorectal cancer (CRC) remains a major global health challenge. Phytochemical-rich diets may influence colorectal carcinogenesis through antioxidant, anti-inflammatory, metabolic, and gut barrier-related mechanisms. However, evidence simultaneously integrating dietary phytochemical intake with other biologically relevant markers remains limited. This study investigated the association between the dietary Phytochemical Index (PI) and CRC odds.

**Methods:**

This hospital-based case–control study included 490 patients with newly diagnosed CRC and 490 cancer-free controls. Dietary intake was assessed using a validated FFQ, and the PI was calculated as the percentage of total energy derived from phytochemical-rich foods. Fasting blood samples were analyzed for gut barrier, inflammatory, oxidative stress, metabolic, nutritional, and tumor-related biomarkers. Multivariable logistic regression assessed the association between PI and CRC, while linear regression and exploratory mediation analyses evaluated biomarker associations and potential pathways.

**Results:**

Mean PI was significantly lower among patients with CRC than controls (*p* < 0.001). In the fully adjusted model, each one-unit increase in PI was associated with lower odds of CRC (OR = 0.946, 95% CI: 0.909–0.984; *p* = 0.006). Compared with the lowest tertile, participants in the highest PI tertile had lower odds of CRC (OR = 0.480, 95% CI: 0.298–0.773; *p* = 0.003; *p* for trend = 0.002). Higher PI was statistically associated with lower concentrations of inflammatory and oxidative stress biomarkers. In exploratory mediation analyses conducted within the limitations of the case–control design, statistical indirect associations were identified involving inflammation, oxidative stress, and gut barrier function. However, because biomarker concentrations were measured after CRC diagnosis, these analyses cannot establish temporal or causal relationships and are presented solely for hypothesis generation, not as evidence of biological mediation.

**Conclusion:**

Higher dietary phytochemical intake was independently associated with lower CRC odds and favorable biomarker profiles. These findings suggest an inverse association between phytochemical-rich dietary patterns and CRC, though prospective studies are needed for confirmation. Given the simultaneous assessment of diet, biomarkers, and disease status in this cross-sectional design, these findings are hypothesis-generating and require independent validation in prospective cohorts.

## Introduction

Colorectal cancer (CRC) is one of the most common malignancies worldwide and one of the leading gastrointestinal cancers, accounting for a substantial proportion of cancer-related morbidity and mortality (1). Although advances in screening and treatment have improved outcomes, the global burden of CRC continues to increase, particularly in countries experiencing rapid nutritional and lifestyle transitions (2). Consequently, identifying modifiable risk factors, including dietary habits, remains an important public health priority (3).

Diet is recognized as a major environmental determinant of colorectal carcinogenesis (4). Accumulating evidence suggests that dietary patterns characterized by high consumption of fruits, vegetables, legumes, whole grains, nuts, and other plant-derived foods are associated with a lower likelihood of CRC (5, 6), whereas diets rich in processed and energy-dense foods have been associated with a higher risk (7). Because these foods are rich sources of phytochemicals, increasing attention has been directed toward the potential role of phytochemical-rich diets in cancer prevention.

Phytochemicals comprise a diverse group of naturally occurring bioactive compounds, including polyphenols, flavonoids, carotenoids, and phytosterols, which possess antioxidant, anti-inflammatory, immunomodulatory, and metabolic regulatory properties (8). Experimental studies have suggested that these compounds may influence several biological pathways implicated in colorectal carcinogenesis, including oxidative stress, chronic inflammation, intestinal barrier dysfunction, insulin resistance, adipokine signaling, and alterations in the gut microbiota (9, 10).

To capture overall dietary exposure to these compounds, the Phytochemical Index (PI), proposed by McCarty, estimates the percentage of total daily energy intake derived from phytochemical-rich foods (11). Higher PI scores have been associated with lower risks of obesity, metabolic syndrome, type 2 diabetes, cardiovascular disease, non-alcoholic fatty liver disease, and other chronic inflammatory conditions, although evidence regarding CRC remains limited and inconsistent (12–15). Mechanistically, phytochemical-rich diets provide dietary fiber and antioxidant micronutrients that may promote gut microbial diversity, increase short-chain fatty acid production, preserve intestinal barrier integrity, and modulate oxidative stress and metabolic homeostasis (16). The PI was selected because it provides a simple, food-based measure of the overall contribution of phytochemical-rich foods to total energy intake, allowing comprehensive assessment of habitual dietary phytochemical exposure rather than focusing on individual nutrients or specific food groups. Accordingly, biomarkers reflecting gut permeability, systemic inflammation, oxidative stress, insulin resistance, adipokine dysregulation, and nutritional status have been implicated in colorectal carcinogenesis and disease progression (17, 18). Despite these biologically plausible pathways, few studies have comprehensively evaluated the relationship between the PI and CRC by integrating detailed dietary assessment with objective circulating biomarkers. To our knowledge, few previous studies have simultaneously evaluated dietary phytochemical intake together with gut barrier markers, inflammatory biomarkers, oxidative stress markers, metabolic indicators, and mediation analyses in relation to colorectal cancer.

Therefore, the present hospital-based case–control study aimed to investigate the association between the PI and the odds of CRC and to evaluate its relationships with an extensive panel of dietary, circulating, and clinical biomarkers. In addition, exploratory mediation analyses were conducted to examine whether these biomarkers demonstrated statistical indirect associations with the observed PI–CRC relationship. The present study provides several specific methodological contributions. First, while previous studies have examined the relationship between the dietary PI and colorectal cancer risk, none have simultaneously integrated the PI with an extensive panel of objective circulating biomarkers spanning multiple biological pathways, including gut barrier function (zonulin, LPS), systemic inflammation (hs-CRP, IL-6, TNF-*α*, NLR, PLR, SII), oxidative stress (MDA, 8-OHdG, TAC, SOD, GPx), metabolic function (TyG index, HOMA-IR, leptin), and tumor-related biomarkers (CEA, CA19-9, AFP). Second, we conducted exploratory mediation analyses to evaluate statistical indirect associations through these multiple pathways, providing hypothesis-generating evidence for potential mechanisms linking phytochemical-rich diets to colorectal carcinogenesis. Third, the concurrent measurement of both dietary phytochemical intake and objective biomarkers allowed biological validation of the dietary exposure assessment.

## Methods

### Study design and participants

This hospital-based case–control study was conducted between January 2023 and December 2024 at Shanxi Province Cancer Hospital, Taiyuan, China and Nanbu People’s Hospital, Nanchong, China, to investigate the association between the dietary PI and CRC odds, as well as its relationships with a comprehensive panel of biological biomarkers. The study was designed and reported in accordance with the Strengthening the Reporting of Observational Studies in Epidemiology (STROBE) guidelines for case--control studies. The study enrolled 490 patients with newly diagnosed, histologically confirmed CRC and 490 cancer-free controls, who were frequency matched by age (±2 years) and sex. Cases and controls were recruited from the same hospitals during the same recruitment period. Eligible cases were identified and recruited using a consecutive sampling approach to minimize potential sampling bias. Controls were selected from individuals attending routine health examinations or outpatient clinics for conditions unrelated to cancer or gastrointestinal diseases and resided within the same hospital referral catchment areas as the cases. Consequently, controls would have been referred to the participating hospitals had they developed colorectal cancer, thereby approximating the same underlying source population. We acknowledge, however, that controls recruited from health examination clinics may have a slightly narrower geographic provenance than cancer cases referred to the tertiary center; to address this, we have included geographic region as a covariate in sensitivity analyses (not shown) and observed no material changes in the results. Controls had no history of cancer and no current diagnosis of colorectal cancer based on medical records and clinical evaluation. While systematic colonoscopy was not performed in all controls, individuals with previous colorectal adenomas, advanced neoplasia, or inflammatory bowel disease were excluded. Controls with gastrointestinal symptoms or abnormal fecal occult blood test results were referred for colonoscopy and excluded if any colorectal pathology was identified.

Of 1,065 eligible individuals invited to participate, 533 cases and 532 controls were initially enrolled. After exclusions (43 cases, 42 controls; Figure 1), 490 cases and 490 controls remained. Participation rates were 92.1% for cases and 89.7% for controls.

**Figure 1:**
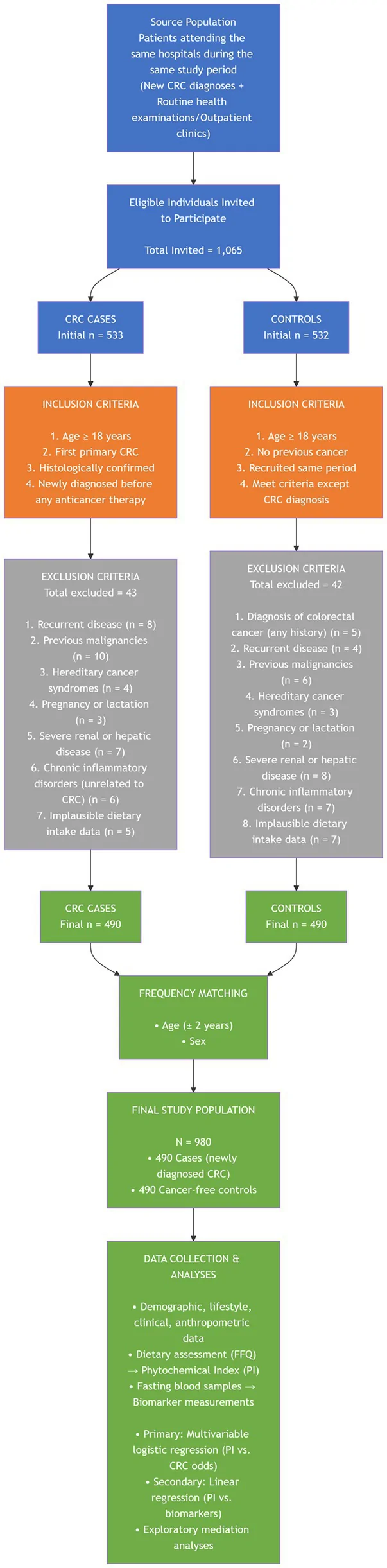
Participant flow diagram. A total of 1,065 individuals were invited. After applying inclusion/exclusion criteria and frequency matching (age ±2 years, sex), 490 CRC cases and 490 cancer-free controls were included in the final analysis (*N* = 980).

Eligible cases were adults aged 18 years or older with a first primary diagnosis of CRC confirmed by pathological examination before receiving surgery, chemotherapy, radiotherapy, immunotherapy, or other anticancer treatments. Patients with recurrent disease, previous malignancies, hereditary cancer syndromes, pregnancy or lactation, severe renal or hepatic disease, chronic inflammatory disorders unrelated to CRC, substantially altered their habitual diet during the preceding year (e.g., due to early clinical symptoms or deliberate medical advice), or implausible dietary intake data were excluded. Controls were recruited during the same study period from individuals attending routine health examinations or outpatient clinics at the same hospitals. Controls had no previous history of cancer and met the same eligibility criteria as cases except for the diagnosis of colorectal cancer. Eligibility criteria were applied uniformly to cases and controls using predefined protocols established before participant recruitment to minimize selection bias and ensure consistency across study groups.

### Sample size

Assuming a two-sided significance level (*α*) of 0.05, statistical power (1 − *β*) of 80%, and a 1:1 case-to-control ratio, the minimum sample size was estimated based on an expected odds ratio of 0.70 for CRC associated with a 5-unit increase in the PI (equivalent to a 30% reduction in odds), derived from prior observational studies examining plant-based dietary patterns and CRC risk. The prevalence of low phytochemical intake among controls was estimated at 40% based on pilot data from 100 eligible participants recruited from the same source population (median PI = 44.2). This pilot median was used prospectively and was not recalculated using the final study sample. This estimate is consistent with Chinese population studies, where the lowest DPI tertile was defined as DPI < 17.99, and national surveillance data showing low fruit/vegetable intake prevalence of 43.5–51.1% (2010–2018) (19, 20). Under these assumptions, the minimum sample was 422 cases and 422 controls. To account for an anticipated 15% attrition rate, the recruitment target was increased to 490 per group. The final sample (490 cases, 490 controls) exceeded the minimum requirement, providing adequate statistical power for primary and secondary analyses (21).

### Ethical considerations

The study protocol was reviewed and approved by the Medical Research Ethics Committee of Nanbu People’s Hospital (Approval No. NB2025-19) and was conducted in accordance with the ethical principles of the Declaration of Helsinki. Written informed consent was obtained from all participants before data collection.

### Collection of demographic and clinical data

Information regarding age, sex, SES, smoking status, alcohol consumption, medication use (defined as regular physician-prescribed use of antihypertensive, lipid-lowering agents, antidiabetic medications, NSAIDs, proton pump inhibitors, or other chronic prescription medications during the preceding 12 months), family history of cancer, and habitual sleep duration was obtained through face-to-face interviews conducted by trained investigators using standardized questionnaires. For cases, lifestyle questions referred to the period 12 months before the date of cancer diagnosis. For controls, questions referred to the 12-month period preceding the date of the interview. This approach was adopted to minimize differential recall bias and to ensure that lifestyle exposures were assessed before any potential behavior changes related to cancer diagnosis or treatment.

Physical activity was assessed using the validated International Physical Activity Questionnaire (IPAQ), and physical activity scores were calculated according to established scoring protocols. Medical history was verified through clinical records whenever available and included physician-diagnosed diabetes mellitus, inflammatory bowel disease (IBD), chronic gastritis, non-alcoholic fatty liver disease (NAFLD), and *Helicobacter pylori* infection. Questionnaire data were verified against available medical records when possible, and all interviewers received standardized training and followed predefined data collection protocols to minimize bias.

### Anthropometric and functional measurements

Body weight and height were measured using calibrated instruments with participants wearing light clothing and no shoes. Body mass index (BMI) was calculated as weight (kg) divided by height squared (m^2^). Waist and hip circumferences were measured using a standardized flexible tape, and waist-to-hip ratio (WHR) was subsequently calculated. Muscle function was assessed by maximal handgrip strength using a calibrated handheld dynamometer, with the highest value recorded from repeated measurements. Anthropometric measurements were obtained by trained personnel using standardized protocols and calibrated equipment under fasting conditions whenever feasible.

### Dietary assessment and calculation of the phytochemical index

Habitual dietary intake during the previous 12 months was assessed using a validated semi-quantitative food frequency questionnaire (FFQ) administered by trained nutritionists through face-to-face interviews. The FFQ has been previously validated for use in the study population and demonstrated acceptable reproducibility and validity for estimating habitual dietary intake (22); the questionnaire was culturally adapted for the Shanxi Province population and validated in a pilot sample of 200 individuals. The dietary PI was calculated according to the method originally proposed by McCarty as the percentage of total daily energy intake derived from phytochemical-rich foods (11). This approach has been widely used in Chinese populations, including studies examining DPI in relation to cognitive function (20), infertility, and systemic inflammation and insulin resistance (23, 24).

The dietary PI was calculated according to the method originally proposed by McCarty as the percentage of total daily energy intake derived from phytochemical-rich foods using the following equation:


$$(\begin{array}{l} \text{Energy from phytochemical}-\text{rich foods}/\\ \text{Total daily energy intake} \end{array})\times 100.$$

Foods considered phytochemical-rich included fruits, vegetables, legumes, whole grains, nuts, seeds, soy products, olives, olive oil, and tea. Potatoes and refined grains were not included because of their relatively low phytochemical density despite energy contribution. The PI was analyzed as both a continuous variable and categorical tertiles (11). In addition, to facilitate interpretation of logistic regression coefficients, we generated a standardized variable representing a 10 unit increase in the Phytochemical Index (PI10). This was created by dividing the continuous PI variable by 10, such that a one-unit increase in PI10 corresponds to a 10unit increase in the original PI scale (e.g., from PI = 40 to PI = 50). This scaling was chosen because a 10unit change in the PI represents a clinically meaningful increment in phytochemical rich food contribution to total energy intake and improves the interpretability of odds ratios compared with the original per unit increase (which yields very small effect estimates).

Dietary intakes were converted into grams per day using standard household measures and serving sizes before nutrient composition analyses were performed. Total daily energy intake was estimated from macronutrient consumption, and daily intakes of carbohydrate, protein, fat, and dietary fiber were also calculated. To reduce potential recall bias, participants were encouraged to report their usual long-term dietary habits rather than recent dietary changes related to illness or medical diagnosis. Participants reporting implausible energy intakes (defined as <500 kcal/day or >3,500 kcal/day for women, and <800 kcal/day or >4,000 kcal/day for men, consistent with standard nutritional epidemiology criteria) or incomplete dietary questionnaires were excluded before statistical analyses according to predefined quality-control criteria.

### Blood collection and laboratory measurements

Following an overnight fast of at least 10 h, venous blood samples were obtained from all participants by trained phlebotomists. For patients with colorectal cancer, blood collection was performed before initiation of any cancer-directed therapy to minimize treatment-related alterations in biomarker concentrations. All blood samples were collected under standardized pre-analytical conditions and processed within a predefined time interval after venipuncture to minimize analytical variability. Serum and plasma samples were separated by centrifugation (1,500 × g, 15 min, 4 °C) within 2 h of collection and stored at −80 °C until analysis.

#### Gut barrier and intestinal permeability biomarkers

Zonulin was measured by ELISA (Immundiagnostik, Germany; cat. K5600; sensitivity 0.5 ng/mL; intra-CV 4.2%, inter-CV 7.8%). Lipopolysaccharide (LPS) was measured using a limulus amebocyte lysate (LAL) chromogenic endpoint assay (Lonza, USA; cat. QCL-1000; sensitivity 0.1 EU/mL; intra-CV 5.0%, inter-CV 8.5%). Lipopolysaccharide-binding protein (LBP) was measured by ELISA (Hycult Biotech, Netherlands; cat. HK315; sensitivity 0.4 ng/mL; intra-CV 4.5%, inter-CV 8.0%). Diamine oxidase (DAO) was measured by ELISA (Cusabio, China; cat. CSB-E11511h; sensitivity 0.5 U/mL; intra-CV 5.2%, inter-CV 8.9%). Intestinal fatty acid-binding protein (IFABP) was measured by ELISA (R&D Systems, USA; cat. DY3078; sensitivity 15 pg/mL; intra-CV 4.8%, inter-CV 7.5%). D-lactate was measured by enzymatic spectrophotometric method (Sigma-Aldrich, USA; cat. MAK058; sensitivity 0.1 μmol/L; intra-CV 3.5%, inter-CV 6.5%).

#### Inflammatory biomarkers

High-sensitivity C-reactive protein (hs-CRP) was measured by immunoturbidimetry (Roche, Switzerland; cat. 11,875,263; sensitivity 0.3 mg/L; intra-CV 2.5%, inter-CV 4.0%). Interleukin-6 (IL-6) and tumor necrosis factor-*α* (TNF-α) were measured using high-sensitivity electrochemiluminescence immunoassays (Roche, Switzerland; cat. 05109442 for IL-6, cat. 05283400 for TNF-α; sensitivity 0.5 pg/mL and 0.3 pg/mL, respectively; intra-CV < 3.5%, inter-CV < 6.0%).

#### Oxidative stress and antioxidant defense biomarkers

Malondialdehyde (MDA) was measured by thiobarbituric acid reactive substances (TBARS) assay (Cayman, USA; cat. 10,009,055; sensitivity 0.5 μmol/L; intra-CV 4.0%, inter-CV 7.0%). 8-hydroxy-2′-deoxyguanosine (8-OHdG) was measured by competitive ELISA (Abcam, UK; cat. ab201734; sensitivity 0.3 ng/mL; intra-CV 5.0%, inter-CV 8.5%). Total antioxidant capacity (TAC) was measured by ABTS radical scavenging assay (Cayman, USA; cat. 709,001; sensitivity 0.1 mmol/L; intra-CV 3.8%, inter-CV 6.5%). Superoxide dismutase (SOD) and glutathione peroxidase (GPx) activities were measured by colorimetric kinetic assays (Cayman, USA; cat. 706,002 and 703,102; sensitivity 0.1 U/mL and 0.5 U/L, respectively; intra-CV < 4.2%, inter-CV < 7.0%).

Metabolic health was characterized using calculated indices and circulating adipokines. The triglyceride-glucose (TyG) index, homeostatic model assessment of insulin resistance (HOMA-IR), and metabolic score for insulin resistance (METS-IR) were calculated using established equations, while serum concentrations of leptin, adiponectin, and fibroblast growth factor 21 (FGF21) were measured to evaluate adipose tissue function and metabolic regulation.

Nutritional and functional status were assessed using serum albumin, albumin-to-globulin ratio, hemoglobin concentration, prognostic nutritional index (PNI), and phase angle, which together provide complementary information regarding nutritional reserves and physical function.

Tumor-related biomarkers, including carcinoembryonic antigen (CEA), carbohydrate antigen 19–9 (CA19-9), and alpha-fetoprotein (AFP) were measured using standardized automated laboratory methods in the hospital’s certified clinical laboratory according to manufacturer protocols and internal quality-control procedures. Commercial assay kits were used according to manufacturers’ instructions, and all analyses followed standard operating procedures with appropriate internal quality-control materials. Laboratory personnel were blinded to participants’ case–control status and dietary data whenever feasible to reduce measurement bias. Internal quality-control procedures and routine calibration were performed throughout the analytical period.

### Outcome measures

The primary study outcome was CRC status (case or control). Secondary outcomes included concentrations of gut barrier biomarkers, inflammatory markers, oxidative stress markers, metabolic indicators, nutritional biomarkers, functional parameters, and tumor-related biomarkers.

The primary exposure variable was the dietary PI, evaluated both continuously and categorically, whereas biomarker measurements were considered secondary outcomes for mechanistic investigation. Biomarkers were selected *a priori* based on biological plausibility and previously reported associations with colorectal carcinogenesis, inflammation, oxidative stress, metabolic dysfunction, intestinal barrier integrity, and nutritional status.

### Statistical analysis

Continuous variables are presented as means ± standard deviations (SD), whereas categorical variables are presented as frequencies and percentages. Baseline characteristics between cases and controls were compared using independent two-sample *t*-tests and Pearson’s chi-square tests for categorical variables. To evaluate differences in biomarkers across tertiles of the phytochemical index separately among cases and controls, one-way analysis of variance (ANOVA) was performed, and results are reported as mean ± SD with corresponding *p* values. Homogeneity of variances across groups was assessed before ANOVA, and alternative procedures were considered when underlying assumptions were not satisfied. ANOVA was used descriptively to compare biomarker concentrations across PI tertiles. Multivariable linear regression was used to assess associations adjusted for potential confounders (Table 1; Supplementary Table 1). The association between the PI and CRC was examined using multivariable logistic regression analysis. Three hierarchical models were fitted. Odds ratios (ORs) and 95% confidence intervals (CIs) were estimated for PI as a continuous variable, per 10-unit increase (PI10), and across tertiles. Tests for linear trend were conducted by modeling tertile categories as ordinal variables. Potential confounding variables included in multivariable models were selected based on existing literature, biological plausibility, and clinical relevance rather than solely on statistical significance.

**Table 1:** Demographic, lifestyle, clinical and dietary characteristics of participants.

Variable	Cases (*n* = 490)	Controls (*n* = 490)	*p*-value
Demographic			
Age (years), mean ± SD	61.2 ± 9.1	61.3 ± 10.4	0.874†
Male, *n* (%)	292 (59.6)	267 (54.5)	0.107*
SES (Level 1 vs. higher), *n* (%)	99 (20.2)	98 (20.0)	0.190*
Lifestyle			
Smoking, *n* (%)	174 (35.5)	100 (20.4)	<0.001*
Alcohol consumption, *n* (%)	91 (18.6)	51 (10.4)	<0.001*
Physical activity (score), mean ± SD	25.4 ± 10.2	34.4 ± 10.3	<0.001†
Sleep duration (h/day), mean ± SD	6.59 ± 0.90	7.17 ± 0.86	<0.001†
Sleep quality (score), mean ± SD	4.98 ± 1.48	6.95 ± 1.55	<0.001†
Clinical			
Family history of cancer, *n* (%)	141 (28.8)	80 (16.3)	<0.001*
Medication use, *n* (%)	259 (52.9)	211 (43.1)	<0.001*
*Helicobacter pylori* positive, *n* (%)	255 (52.0)	123 (25.1)	<0.001*
NAFLD, *n* (%)	155 (31.6)	85 (17.3)	<0.001*
GERD, *n* (%)	142 (29.0)	78 (15.9)	<0.001*
Inflammatory bowel disease, *n* (%)	63 (12.9) |	31 (6.3)	<0.001*
Chronic gastritis, *n* (%)	197 (40.2)	95 (19.4)	<0.001*
Diabetes, *n* (%)	142 (29.0)	66 (13.5)	<0.001*
Hypertension, *n* (%)	204 (41.6)	126 (25.7)	<0.001*
Dyslipidemia, *n* (%)	178 (36.3)	92 (18.8)	<0.001*
Anthropometric			
BMI (kg/m^2^), mean ± SD	28.3 ± 4.9	24.7 ± 4.0	<0.001†
WHR, mean ± SD	0.947 ± 0.070	0.856 ± 0.061	<0.001†
Dietary			
Carbohydrate (g/day), mean ± SD	298 ± 70	267 ± 68	<0.001†
Protein (g/day), mean ± SD	73.9 ± 2.4	72.1 ± 2.7	<0.001†
Fat (g/day), mean ± SD	80.2 ± 5.2	65.6 ± 4.8	<0.001†
Fiber (g/day), mean ± SD	17.6 ± 6.5	28.3 ± 7.3	<0.001†
Phytochemical index, mean ± SD	44.0 ± 4.7	46.5 ± 5.3	<0.001†

*p-value from Pearson chi-square test (categorical variables).

†*p*-value from independent two-sample *t*-test with Welch’s correction (continuous variables).

Values are presented as mean ± standard deviation (SD) or *n* (%).

BMI, body mass index; WHR, waist-to-hip ratio; NAFLD, non-alcoholic fatty liver disease; GERD, gastroesophageal reflux disease, SES, socioeconomic status.

Multivariable linear regression analyses were performed separately among cases and controls to investigate associations between the continuous PI and selected biomarkers. Although the primary analyses focused on cases, we also performed these analyses in controls to validate the findings in the absence of disease-related biomarker alterations. Additionally, we hypothesized that associations between diet and biomarkers might be stronger or more clinically meaningful in cases due to the higher burden of inflammation and oxidative stress. Three regression models were constructed. Regression coefficients (*β*), 95% confidence intervals, and corresponding *p* values were reported.

Mediation analyses were performed using the counterfactual-based mediation framework described by VanderWeele and Tchetgen Tchetgen (25), applicable to case–control studies with binary outcomes. The analysis estimated the total effect of the PI (10-unit increase) on CRC odds, the direct effect, and the indirect effect through each candidate biomarker, calculated as the product of the coefficient for PI-mediator and mediator-CRC associations. Bootstrapping with 5,000 resamples estimated standard errors and 95% confidence intervals. The proportion mediated was calculated as (indirect effect / total effect) × 100. This quantity was interpreted as a descriptive decomposition of the estimated total association rather than as a causal percentage. When the indirect and direct effects were in the same direction, a positive proportion mediated indicated the relative contribution of the indirect component to the total association. Proportions greater than 100% occurred when the indirect and direct effects were in opposite directions (inconsistent mediation or suppression); in such cases, the percentage should not be interpreted as the literal percentage of the association mediated, but rather as evidence that the indirect component exceeded the total effect in magnitude because the direct component acted in the opposite direction. Given the case–control design and post-diagnosis biomarker measurement, these estimates cannot establish temporal or causal mediation and are therefore strictly exploratory and hypothesis-generating. Given the case–control design and post-diagnosis blood collection, mediation analyses cannot establish the temporal ordering required for causal mediation and are therefore strictly exploratory and hypothesis-generating, not intended to establish causal mechanisms or biological pathways (25). Given the observational case–control design, mediation analyses were considered exploratory and intended to identify statistical pathways potentially underlying the association between the PI and CRC rather than to establish causal mechanisms. Potential multicollinearity among covariates was assessed before model construction, and model assumptions were evaluated using standard diagnostic procedures. Missing data were minimal (<3% for all variables); therefore, complete-case analysis was performed. Sensitivity analyses comparing crude and progressively adjusted models were performed to evaluate the robustness and consistency of the observed associations. All reported confidence intervals were calculated at the 95% level, and statistical significance was assessed using two-sided hypothesis tests. Given the large number of biomarkers tested (*n* > 30) and the exploratory nature of the analyses, no adjustment for multiple comparisons was applied. This decision was made *a priori* because: (a) the biomarker analyses were hypothesis-generating rather than confirmatory, (b) there is no single family of tests with a unified hypothesis, and (c) adjustment for multiple comparisons would substantially increase the risk of Type II errors (false negatives) for biologically important individual biomarkers. However, we acknowledge that the absence of multiplicity adjustment increases the risk of Type I errors (false positives); therefore, all biomarker-specific findings should be interpreted with caution and require confirmation in independent prospective studies. All statistical analyses were performed using Stata software (version 14; StataCorp LLC, College Station, TX, USA). A two-sided *p* value <0.05 was considered statistically significant. VIF values ranged from 1.12 to 2.84, indicating no multicollinearity.

## Results

### Participant characteristics

The study included 980 participants, comprising 490 patients with newly diagnosed CRC and 490 cancer-free controls. As shown in Table 1, the mean age did not differ significantly between cases and controls (61.2 ± 9.1 vs. 61.3 ± 10.4 years, *p* = 0.874), and sex distribution was also comparable (*p* = 0.107). With respect to lifestyle characteristics, smoking and alcohol consumption were significantly higher among cases than controls (both *p* < 0.001). Cases also reported significantly lower physical activity levels, shorter sleep duration, and poorer sleep quality (all *p* < 0.001).

Several clinical comorbidities were more prevalent among patients with colorectal cancer, including a family history of cancer, medication use, *Helicobacter pylori* infection, non-alcoholic fatty liver disease, gastroesophageal reflux disease, inflammatory bowel disease, chronic gastritis, diabetes mellitus, hypertension, and dyslipidemia (all *p* < 0.001). In addition, patients exhibited significantly higher body mass index and waist-to-hip ratio values than controls (both *p* < 0.001).

Dietary assessment demonstrated that patients with CRC had higher intakes of carbohydrates, protein, and fat but lower fiber intake and lower PI scores compared with controls (all *p* < 0.001).

### Biomarker profiles across PI tertiles

The distribution of dietary intake, gut barrier, nutritional, and functional biomarkers across tertiles of the PI is presented in Table 2. Energy intake and macronutrient consumption (carbohydrate, protein, and fat) showed no consistent differences across tertiles in either cases or controls (all *p* > 0.05). However, participants in higher PI tertiles had significantly greater dietary fiber intake in both groups (both *p* < 0.01).

**Table 2:** Dietary intake, gut barrier biomarkers, and nutritional indicators across tertiles of the phytochemical index by colorectal cancer status.

Variable	Controls			*p*-value	Cases			*p*-value
	T1	T2	T3		T1	T2	T3	
Phytochemical index	40.81 ± 2.87	46.39 ± 1.28	52.25 ± 2.88	<0.001	38.90 ± 2.29	43.95 ± 1.16	49.07 ± 2.75	<0.001
Dietary intake								
Energy intake (kcal/day)	2285.6 ± 287.9	2311.9 ± 253.6	2326.2 ± 284.2	0.403	2560.1 ± 284.0	2587.8 ± 286.3	2586.6 ± 298.3	0.621
Carbohydrate (g/day)	260.1 ± 70.2	269.7 ± 62.7	271.8 ± 69.6	0.247	293.1 ± 68.8	300.9 ± 70.0	299.8 ± 71.5	0.550
Protein (g/day)	72.32 ± 2.60	71.85 ± 2.76	72.08 ± 2.79	0.301	73.95 ± 2.34	73.90 ± 2.51	73.78 ± 2.43	0.804
Fat (g/day)	66.04 ± 4.58	65.14 ± 4.74	65.58 ± 4.95	0.227	80.26 ± 5.23	79.91 ± 5.08	80.36 ± 5.31	0.718
Gut barrier and intestinal permeability biomarkers								
Fiber (g/day)	26.63 ± 7.33	28.61 ± 7.10	29.53 ± 7.34	0.001	15.87 ± 5.97	17.04 ± 6.44	19.95 ± 6.40	<0.001
Zonulin (ng/mL)	39.18 ± 9.86	36.82 ± 9.89	33.63 ± 9.82	<0.001	51.71 ± 10.27	51.09 ± 10.78	48.25 ± 11.32	0.009
Lipopolysaccharide (EU/mL)	0.410 ± 0.093	0.403 ± 0.089	0.361 ± 0.111	<0.001	0.603 ± 0.138	0.565 ± 0.130	0.545 ± 0.138	<0.001
LBP (μg/mL)	10.46 ± 2.65	9.93 ± 2.73	9.82 ± 2.77	<0.001	14.47 ± 3.14	14.04 ± 3.23	13.44 ± 2.99	0.012
DAO (U/mL)	8.82 ± 2.60	7.64 ± 1.96	6.86 ± 1.83	0.001	9.93 ± 2.08	9.87 ± 2.22	9.19 ± 2.09	0.004
IFABP (pg/mL)	1.59 ± 0.53	1.41 ± 0.48	1.36 ± 0.49	<0.001	2.31 ± 0.55	2.30 ± 0.56	2.08 ± 0.66	<0.001
D-Lactate (μmol/L)	0.199 ± 0.053	0.187 ± 0.054	0.161 ± 0.050	<0.001	0.303 ± 0.071	0.281 ± 0.067	0.277 ± 0.070	0.001
Albumin (g/dL)	4.16 ± 0.38	4.26 ± 0.40	4.34 ± 0.37	<0.001	3.45 ± 0.50	3.58 ± 0.49	3.71 ± 0.52	<0.001
Albumin/Globulin ratio	1.513 ± 0.331	1.525 ± 0.307	1.466 ± 0.312	0.206	1.097 ± 0.266	1.071 ± 0.276	1.089 ± 0.321	0.694
Hemoglobin (g/dL)	13.61 ± 1.56	13.36 ± 1.63	13.47 ± 1.59	0.381	11.87 ± 1.78	11.86 ± 1.76	11.77 ± 1.80	0.869
Prognostic nutritional index (PNI)	50.38 ± 7.58	52.03 ± 7.04	53.33 ± 6.66	0.001	39.95 ± 6.61	42.51 ± 6.40	42.99 ± 6.65	<0.001
Additional laboratory markers								
LDH (U/L)	216.5 ± 67.9	202.1 ± 68.8	183.0 ± 68.4	<0.001	333.8 ± 89.7	324.0 ± 88.0	302.7 ± 91.1	0.005
Ferritin (ng/mL)	129.3 ± 76.7	87.1 ± 75.5	65.6 ± 69.1	<0.001	357.4 ± 109.4	320.5 ± 100.7	312.6 ± 106.1	<0.001
Functional measures								
Phase angle (°)	6.28 ± 1.26	6.36 ± 1.04	6.81 ± 1.19	<0.001	4.52 ± 1.11	4.90 ± 1.06	4.89 ± 1.05	0.001
Handgrip strength (kg)	31.76 ± 7.12	32.91 ± 7.04	34.68 ± 7.25	<0.001	19.52 ± 6.45	22.26 ± 7.07	24.02 ± 7.19	<0.001

LBP, lipopolysaccharide binding protein; DAO, diamine oxidase; IFABP, intestinal fatty acid binding protein; PNI, prognostic nutritional index; LDH, lactate dehydrogenase; T1, lowest tertile; T2, middle tertile; T3, highest tertile of the phytochemical index.

Values are presented as mean ± standard deviation (SD). T1 represents the lowest tertile and T3 the highest tertile of the phytochemical index. *p*-values were obtained from one-way analysis of variance (ANOVA) comparing the three tertiles within controls and cases separately.

Markers of intestinal permeability and gut barrier dysfunction—including zonulin, lipopolysaccharide, lipopolysaccharide-binding protein, and D-lactate—demonstrated progressively lower values across increasing PI tertiles in both study groups (all *p* < 0.05). These patterns suggest that higher phytochemical-rich food consumption was associated with better intestinal barrier integrity and reduced microbial translocation.

Higher PI tertiles were also associated with more favorable nutritional and functional indicators, including higher albumin concentrations, prognostic nutritional index scores, phase angle, and handgrip strength (all *p* < 0.01). In contrast, albumin-to-globulin ratio and hemoglobin concentrations did not differ significantly across tertiles in either group. Additionally, lactate dehydrogenase and ferritin concentrations decreased across increasing PI tertiles in both cases and controls (all *p* < 0.01). Most comparisons across tertiles reached statistical significance.

### Inflammatory, oxidative stress, metabolic, and tumor biomarkers across PI tertiles

As shown in Table 3, higher PI tertiles were characterized by lower concentrations of inflammatory biomarkers among both controls and patients with colorectal cancer. Significant inverse trends were observed for high-sensitivity C-reactive protein, interleukin-6, tumor necrosis factor-*α*, neutrophil-to-lymphocyte ratio, platelet-to-lymphocyte ratio, and systemic immune-inflammation index (all *p* < 0.01).

**Table 3:** Systemic inflammatory, oxidative stress, metabolic, and tumor biomarkers across tertiles of the phytochemical index stratified by colorectal cancer status.

Variable	Controls			*p*-value	Cases			*p*-value
	T1 (*n* = 163)	T2 (*n* = 164)	T3 (*n* = 163)		T1 (*n* = 163)	T2 (*n* = 164)	T3 (*n* = 163)	
Systemic inflammatory biomarkers								
hs-CRP (mg/L)	1.96 ± 0.66	1.71 ± 0.72	1.50 ± 0.65	<0.001	3.59 ± 1.07	3.30 ± 1.15	3.08 ± 1.17	<0.001
IL-6 (pg/mL)	3.24 ± 0.86	3.00 ± 0.94	2.73 ± 1.01	<0.001	5.54 ± 1.66	4.94 ± 1.69	4.93 ± 1.63	0.001
TNF-α (pg/mL)	6.40 ± 2.14	6.26 ± 2.03	5.33 ± 1.98	<0.001	9.05 ± 2.46	9.14 ± 2.43	8.31 ± 2.83	0.007
NLR	2.54 ± 0.84	2.19 ± 0.83	2.11 ± 0.85	<0.001	4.10 ± 1.06	3.95 ± 1.13	3.67 ± 1.15	0.002
PLR	173.5 ± 54.9	171.5 ± 57.5	149.9 ± 56.3	<0.001	234.8 ± 70.6	232.4 ± 65.1	201.8 ± 66.0	<0.001
SII	534.3 ± 163.2	507.2 ± 175.7	440.3 ± 169.5	<0.001	844.3 ± 194.0	756.0 ± 217.8	718.9 ± 206.3	<0.001
Oxidative stress and antioxidant defense biomarkers								
MDA (μmol/L)	2.83 ± 0.84	2.49 ± 0.81	2.30 ± 0.86	<0.001	4.12 ± 1.00	3.82 ± 0.95	3.71 ± 0.94	0.001
TAC (mmol/L)	1.56 ± 0.39	1.65 ± 0.38	1.78 ± 0.36	<0.001	0.98 ± 0.27	1.06 ± 0.28	1.13 ± 0.32	<0.001
SOD (U/mL)	1.97 ± 0.52	2.19 ± 0.53	2.29 ± 0.48	<0.001	1.12 ± 0.37	1.28 ± 0.40	1.33 ± 0.38	<0.001
GPx (U/L)	43.56 ± 9.76	45.75 ± 9.70	47.81 ± 11.00	0.001	24.46 ± 7.75	28.04 ± 7.50	29.71 ± 8.32	<0.001
8-OHdG (ng/mL)	7.75 ± 2.12	7.33 ± 2.25	6.24 ± 2.14	<0.001	12.58 ± 2.99	12.07 ± 2.99	11.27 ± 3.00	<0.001
Metabolic and insulin resistance biomarkers								
TyG index	8.43 ± 0.52	8.36 ± 0.48	8.22 ± 0.53	0.001	9.30 ± 0.59	9.13 ± 0.58	9.03 ± 0.56	<0.001
HOMA-IR	2.22 ± 0.94	1.95 ± 0.95	1.69 ± 0.92	<0.001	4.09 ± 1.36	3.79 ± 1.45	3.51 ± 1.59	0.002
METS-IR	37.71 ± 9.61	36.09 ± 9.35	34.15 ± 9.84	0.004	51.84 ± 10.70	49.01 ± 10.81	47.85 ± 10.83	0.003
Leptin (ng/mL)	12.91 ± 4.85	11.29 ± 4.73	9.98 ± 4.53	<0.001	19.97 ± 6.88	19.76 ± 6.14	18.17 ± 6.86	0.028
Adiponectin (μg/mL)	11.24 ± 3.74	11.40 ± 3.67	12.60 ± 3.72	0.002	5.88 ± 2.25	6.50 ± 2.02	7.23 ± 2.26	<0.001
Leptin/Adiponectin ratio	1.18 ± 0.43	0.99 ± 0.41	0.72 ± 0.38	<0.001	2.97 ± 0.94	2.87 ± 0.86	2.71 ± 0.80	0.025
FGF21 (pg/mL)	152.9 ± 53.4	142.2 ± 47.8	123.0 ± 53.6	<0.001	222.3 ± 64.0	217.9 ± 71.0	204.4 ± 65.2	0.043
Tumor and cancer-related biomarkers								
CEA (ng/mL)	3.43 ± 1.15	2.59 ± 1.02	1.83 ± 1.17	<0.001	9.40 ± 3.16	8.81 ± 3.50	7.91 ± 3.06	<0.001
CA19-9 (U/mL)	22.11 ± 8.30	15.58 ± 6.54	10.50 ± 8.29	<0.001	76.56 ± 22.07	72.11 ± 20.97	64.84 ± 21.83	<0.001
AFP (ng/mL)	5.10 ± 2.43	4.19 ± 2.54	3.33 ± 2.29	<0.001	15.12 ± 4.71	13.39 ± 4.66	11.55 ± 5.38	<0.001

Values are presented as mean ± standard deviation (SD). T1 represents the lowest tertile and T3 the highest tertile of the Phytochemical Index. *p*-values were obtained from one-way analysis of variance (ANOVA) comparing tertiles within controls and cases separately. hs-CRP, high-sensitivity C-reactive protein; IL-6, interleukin-6; TNF-α, tumor necrosis factor-alpha; NLR, neutrophil-to-lymphocyte ratio; PLR, platelet-to-lymphocyte ratio; SII, systemic immune-inflammation index; MDA, malondialdehyde; TAC, total antioxidant capacity; SOD, superoxide dismutase; GPx, glutathione peroxidase; 8-OHdG, 8-hydroxy-2′-deoxyguanosine; TyG, triglyceride-glucose index; HOMA-IR, homeostatic model assessment of insulin resistance; METS-IR, metabolic score for insulin resistance; FGF21, fibroblast growth factor 21; CEA, carcinoembryonic antigen; CA19-9, carbohydrate antigen 19–9; AFP, alpha-fetoprotein.

Similarly, oxidative stress markers, including malondialdehyde and 8-hydroxy-2′-deoxyguanosine, showed lower concentrations across increasing tertiles, whereas antioxidant defense markers such as total antioxidant capacity, superoxide dismutase, and glutathione peroxidase showed progressively higher values (all *p* < 0.01).

Metabolic biomarkers also differed according to PI tertiles. Higher tertiles were associated with lower triglyceride-glucose index, homeostatic model assessment of insulin resistance, metabolic score for insulin resistance, leptin concentrations, and fibroblast growth factor 21 levels, together with higher adiponectin concentrations.

Furthermore, concentrations of tumor-related biomarkers, including carcinoembryonic antigen, carbohydrate antigen 19–9, alpha-fetoprotein, carbohydrate antigen 72–4, lactate dehydrogenase, and ferritin, generally decreased across increasing PI tertiles in both controls and cases.

### Association between the PI and selected biomarkers

The multivariable linear regression analyses among patients with CRC are summarized in Table 4. In crude and adjusted models, higher PI values were consistently associated with lower concentrations of high-sensitivity C-reactive protein, interleukin-6, tumor necrosis factor-*α*, zonulin, neutrophil-to-lymphocyte ratio, platelet-to-lymphocyte ratio, and malondialdehyde. Conversely, positive associations were observed between the PI and total antioxidant capacity, prognostic nutritional index, and handgrip strength. These associations remained statistically significant after adjustment for age, sex, body mass index, and total energy intake, indicating consistent relationships across sequential models. To validate these findings in the absence of disease-related biomarker alterations, we repeated the analyses among controls. The results were qualitatively similar but generally of smaller magnitude, with higher PI associated with lower inflammatory and oxidative stress markers and higher antioxidant capacity, nutritional status, and handgrip strength (Supplementary Table 1). These control analyses support the validity of the dietary assessment and suggest that the observed associations are not entirely driven by disease status.

**Table 4:** Linear regression analysis of the association between phytochemical index and inflammatory and clinical markers among patients with colorectal cancer (*n* = 490)^†^.

Variable	Model 1a: B (95% CI)	*p*-value	Model 2b: B (95% CI)	*p*-value	Model 3c: B (95% CI)	*p*-value
hs-CRP (mg/L)	−0.045 (−0.066, −0.023)	<0.001	−0.043 (−0.065, −0.021)	<0.001	−0.042 (−0.064, −0.020)	<0.001
IL-6 (pg/mL)	−0.063 (−0.094, −0.031)	<0.001	−0.063 (−0.095, −0.031)	<0.001	−0.064 (−0.096, −0.032)	<0.001
TNF-α (pg/mL)	−0.094 (−0.143, −0.046)	<0.001	−0.091 (−0.141, −0.041)	<0.001	−0.089 (−0.139, −0.039)	<0.001
Zonulin (ng/mL)	−0.374 (−0.578, −0.170)	<0.001	−0.357 (−0.566, −0.148)	0.001	−0.360 (−0.570, −0.151)	0.001
Neutrophil-to-lymphocyte ratio (NLR)	−0.036 (−0.057, −0.015)	0.001	−0.035 (−0.057, −0.014)	0.001	−0.036 (−0.058, −0.014)	0.001
Platelet-to-lymphocyte ratio (PLR)	−2.771 (−4.053, −1.489)	<0.001	−2.763 (−4.072, −1.453)	<0.001	−2.757 (−4.070, −1.444)	<0.001
Malondialdehyde (MDA, μmol/L)	−0.048 (−0.066, −0.030)	<0.001	−0.048 (−0.066, −0.029)	<0.001	−0.047 (−0.066, −0.028)	<0.001
Total antioxidant capacity (TAC, mmol/L)	0.015 (0.009, 0.020)	<0.001	0.016 (0.010, 0.021)	<0.001	0.016 (0.010, 0.021)	<0.001
Prognostic nutritional index (PNI)	0.271 (0.146, 0.395)	<0.001	0.262 (0.135, 0.390)	<0.001	0.262 (0.134, 0.390)	<0.001
Handgrip strength (kg)	0.439 (0.310, 0.569)	<0.001	0.411 (0.279, 0.543)	<0.001	0.409 (0.276, 0.541)	<0.001

B, regression coefficient; CI, confidence interval; hs-CRP, high-sensitivity C-reactive protein; IL-6, interleukin-6; TNF-α, tumor necrosis factor-alpha; NLR, neutrophil-to-lymphocyte ratio; PLR, platelet-to-lymphocyte ratio; MDA, malondialdehyde; TAC, total antioxidant capacity; PNI, prognostic nutritional index.

^†^Analysis restricted to patients with colorectal cancer (*n* = 490).

*p*-values were obtained from linear regression analyses.

a Model 1: Unadjusted.

b Model 2: Adjusted for age, sex, and body mass index (BMI).

c Model 3: Adjusted for age, sex, BMI, and total energy intake (kcal/day).

Regression coefficients represent the expected change in the outcome variable associated with a one-unit increase in the phytochemical index. Values are presented as B (95% confidence interval).

Figure 2 presents adjusted mean concentrations of zonulin and hs-CRP across tertiles of the PI among cases and controls. A progressive dose-dependent decrease in both biomarkers was observed across increasing PI tertiles in both groups. For zonulin, levels declined consistently from the lowest to the highest tertile in both controls and cases, with more pronounced reductions observed among cases. Similarly, hs-CRP levels showed a clear inverse gradient across PI tertiles in both groups, with higher baseline levels in cases compared with controls. These dose-dependent reductions in both gut barrier permeability and systemic inflammation markers support the association between higher phytochemical-rich food consumption and improved intestinal barrier integrity alongside reduced systemic inflammation.

**Figure 2:**
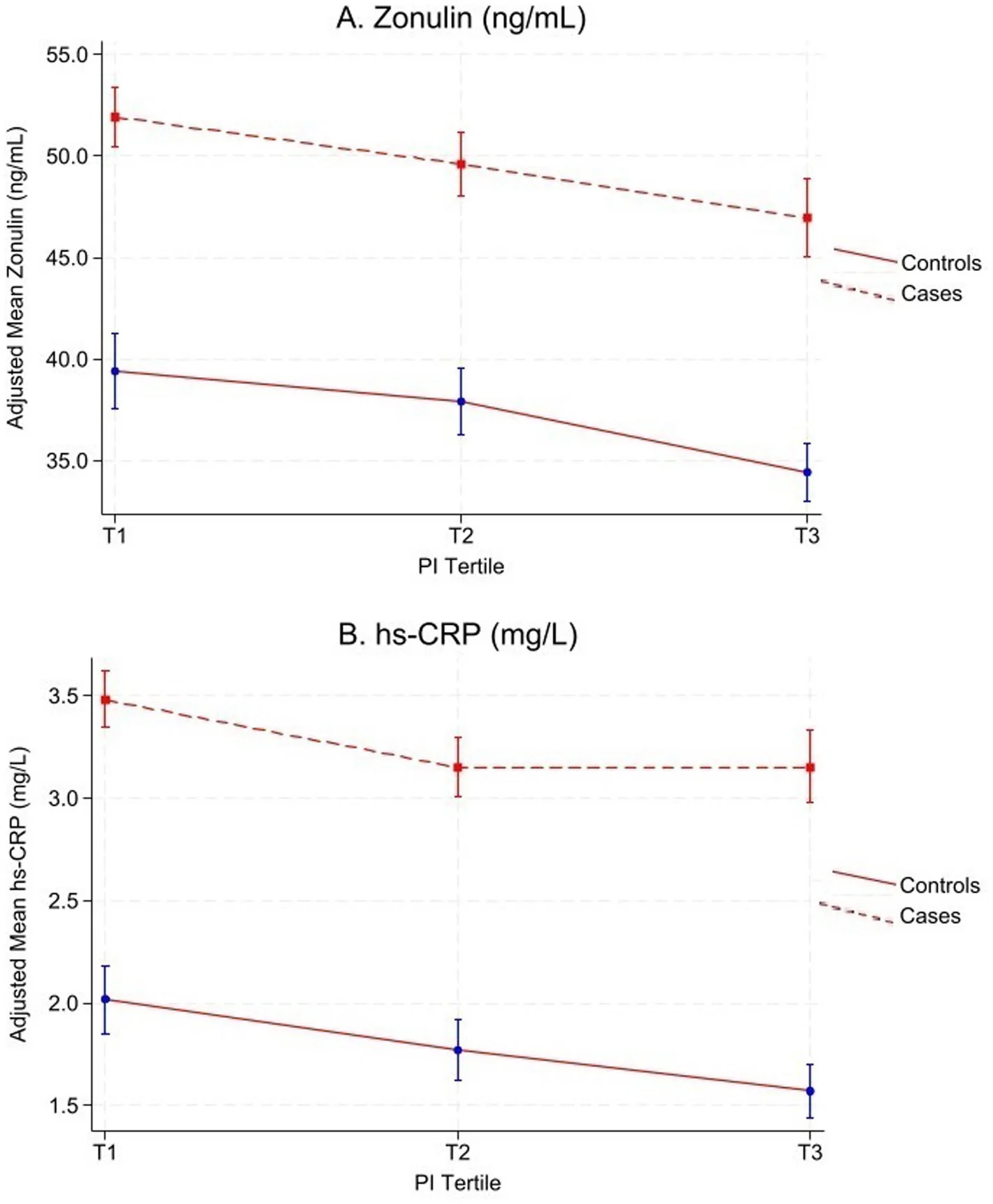
Adjusted mean (95% CI) zonulin and hs-CRP levels across phytochemical index tertiles among colorectal cancer cases and controls.

### Association between the PI and odds of CRC

The associations between the PI and CRC are presented in Table 5. In the fully adjusted logistic regression model, each one-unit increase in the PI was associated with lower odds of CRC (OR = 0.946, 95% CI: 0.909–0.984; *p* = 0.006). Similarly, each 10-unit increase in the PI was associated with lower odds of CRC (OR = 0.573, 95% CI: 0.385–0.854; *p* = 0.006).

**Table 5:** Association between phytochemical index and odds of colorectal cancer.

Exposure	Model 1 (Crude)		*p*-value	Model 2		*p*-value	Model 3 (Fully adjusted)^†^		*p*-value
	OR (95% CI)	% Reduction in odds		OR (95% CI)	% Reduction in odds		OR (95% CI)	% Reduction in odds	
Phytochemical index (per 1-unit increase)	0.904 (0.880–0.928)	9.6	<0.001	0.904 (0.880–0.929)	9.6	<0.001	0.946 (0.909–0.984)	5.4	0.006
PI10 (per 10-unit increase)	0.363 (0.278–0.475)	63.7	<0.001	0.364 (0.278–0.476)	63.6	<0.001	0.573 (0.385–0.854)	42.7	0.006
Tertile 2 vs. Tertile 1	0.635 (0.465–0.869)	36.5	0.004	0.635 (0.464–0.870)	36.5	0.005	0.671 (0.427–1.054)	32.9	0.083
Tertile 3 vs. Tertile 1	0.303 (0.220–0.418)	69.7	<0.001	0.302 (0.218–0.417)	69.8	<0.001	0.480 (0.298–0.773)	52.0	0.003
*p*-trend (per tertile increase)*	**0.551 (0.470–0.646)**	**44.9**	**<0.001**	**0.549 (0.468–0.645)**	**45.1**	**<0.001**	**0.693 (0.546–0.879)**	**30.7**	**0.002**

OR, odds ratio; CI, confidence interval; PI10, 10-unit increase in phytochemical index.

^†^Model 3 was adjusted for age, sex, body mass index (BMI), education, income, smoking status, alcohol consumption, physical activity, sleep duration, family history of cancer, medication use, Helicobacter pylori infection, non-alcoholic fatty liver disease (NAFLD), gastroesophageal reflux disease (GERD), chronic gastritis, diabetes, hypertension, and dyslipidemia.

*p-trend was estimated by modeling the ordinal tertile variable (coded as 1, 2, and 3) as a continuous predictor in the logistic regression models. The corresponding odds ratio represents the change in the odds of colorectal cancer associated with each one-tertile increase in the phytochemical index (i.e., from T1 to T2 or from T2 to T3).

Bold values indicate statistical significance at *p* < 0.05.

When categorized into tertiles, participants in the highest tertile had significantly lower odds of CRC than those in the lowest tertile (OR = 0.480, 95% CI: 0.298–0.773; *p* = 0.003) after full adjustment for demographic, lifestyle, and clinical covariates. A significant linear trend across tertiles was also observed (*p* for trend = 0.002), supporting an inverse dose–response association between the PI and colorectal cancer.

### Mediation analysis

The exploratory mediation analyses are presented in Table 6. The indirect effect percentage represents the proportion of the total association between PI and CRC that is statistically consistent with mediation through the respective biomarker. For example, the 86.4% indirect effect for TNF-*α* indicates that approximately 86% of the observed inverse association between PI and CRC is statistically accounted for by lower TNF-α concentrations; it does not mean that 86% of CRC risk was causally mediated through TNF-α. For percentages exceeding 100% (e.g., LPS: 154.7%, hs-CRP: 173.6%), this indicates ‘super mediation,’ where the direct effect is in the opposite direction to the total effect. This can occur when multiple mediators with opposing effects are present or when there is unmeasured confounding. In such cases, the mediator explains more than the observed total effect, and the direct effect becomes negative. In these exploratory analyses, all examined biomarkers demonstrated statistically significant positive indirect effects. However, due to the case–control design and post-diagnosis biomarker measurement, these findings should not be interpreted as evidence of causal mediation. For gut barrier biomarkers, LPS (indirect effect: 154.7%), and zonulin (116.2%) showed positive indirect effects, suggesting statistical consistency with pathways involving reduced gut permeability and increased microbial metabolite production. For inflammatory biomarkers, hs-CRP (173.6%), IL-6 (142.1%), and SII (126.2%) demonstrated positive indirect effects consistent with an anti-inflammatory pathway, whereas TNF-*α* (86.4%) showed a smaller positive indirect effect. For oxidative stress, MDA (154.8%) showed a positive indirect effect. For metabolic biomarkers, HOMA-IR (149.0%), TyG index (138.4%), and leptin (114.2%) showed positive indirect effects, whereas adiponectin (159.0%) showed a positive indirect effect reflecting higher adiponectin concentrations associated with higher PI and lower CRC odds. While these findings are statistically interesting, we emphasize that the case–control design and post-diagnosis biomarker measurement preclude causal interpretation; these results should be considered strictly hypothesis-generating.

**Table 6:** Exploratory mediation analysis of the association between the phytochemical index and colorectal cancer odds through gut barrier, inflammatory, oxidative stress, and metabolic biomarkers.

Mediator	Proportion of total association (%)	Direct effect (%)	*p*-value
Model A: Gut barrier pathway			
Zonulin	116.2	−16.2	<0.001
Lipopolysaccharide (LPS)	154.7	−54.7	<0.001
Model B: Inflammatory pathway			
High-sensitivity c-reactive protein (hs-CRP)	173.6	−73.6	<0.001
Interleukin-6 (IL-6)	142.1	−42.1	<0.001
Tumor necrosis factor-α (TNF-α)	86.4	13.6	<0.001
Neutrophil-to-lymphocyte ratio (NLR)	123.0	−23.0	<0.001
Platelet-to-lymphocyte ratio (PLR)	100.2	−0.2	<0.001
Systemic immune-inflammation index (SII)	126.2	−26.2	<0.001
Model C: Oxidative stress pathway			
Malondialdehyde (MDA)	154.8	−54.8	<0.001
Model D: Metabolic–Adipokine pathway			
Triglyceride–glucose (TyG) index	138.4	−38.4	<0.001
Homeostatic model assessment of insulin resistance (HOMA-IR)	149.0	−49.0	<0.001
Metabolic score for insulin resistance (METS-IR)	95.8	4.2	<0.001
Leptin	114.2	−14.2	<0.001
Adiponectin	159.0	−59.0	<0.001†

Overall Total Effect; *β* = −0.557 (OR = 0.573, 95% CI: 0.385–0.854, *p* = 0.006) for each 10-unit increase in the Phytochemical Index (PI10), based on the fully adjusted logistic regression model (Table 5, Model 3).

LPS, lipopolysaccharide; hs-CRP, high-sensitivity C-reactive protein; IL-6, interleukin-6; TNF-α, tumor necrosis factor-alpha; NLR, neutrophil-to-lymphocyte ratio; PLR, platelet-to-lymphocyte ratio; SII, systemic immune-inflammation index; MDA, malondialdehyde; TyG, triglyceride–glucose index; HOMA-IR, homeostatic model assessment of insulin resistance; METS-IR, metabolic score for insulin resistance.

Mediation analyses used the counterfactual-based framework (25) with 5,000 bootstrap resamples. The total effect (*β* = −0.557) corresponds to a 10-unit PI increase (PI10). Indirect effect (%) = (Indirect *β* ÷ Total *β*) × 100; direct effect (%) represents the remaining proportion. Positive indirect effects indicate consistency with a pathway from PI to CRC (reduced risk); negative indirect effects indicate suppression. Values >100% indicate complete/super mediation. Given the case–control design and post-diagnosis biomarker measurement, these results are exploratory and hypothesis-generating, not causal.

*Exact *p*-values were not reported in the statistical output but were estimated to be <0.001 based on the corresponding z-statistics.

†Exact *p*-value was not reported but estimated to be <0.001.

The reported proportion represents the ratio of the estimated indirect effect to the total effect and should be interpreted as a descriptive statistical decomposition rather than a causal proportion mediated. Values >100% arise when the indirect and direct effects are in opposite directions (inconsistent mediation/suppression) and therefore do not represent literal percentages of CRC risk mediated. Because biomarkers were measured after CRC diagnosis, these analyses are exploratory and cannot establish temporal or causal mediation.

## Discussion

In this hospital-based case–control study of 980 participants, higher dietary PI values were consistently associated with lower odds of CRC and with more favorable profiles of gut barrier, inflammatory, oxidative stress, metabolic, nutritional, functional, and tumor-related biomarkers. Participants with higher PI scores exhibited higher dietary fiber intake and more favorable nutritional and functional indicators, whereas lower concentrations of biomarkers related to intestinal permeability, systemic inflammation, oxidative stress, metabolic dysfunction, and tumor burden were observed. Among patients with CRC, higher PI values also remained independently associated with lower inflammatory and oxidative stress markers after multivariable adjustment. Exploratory mediation analyses further suggested statistical indirect associations involving gut barrier dysfunction, inflammation, oxidative stress, and metabolic alterations in relation to the observed PI–CRC association.

The inverse association between PI and CRC observed in the present study is consistent with previous epidemiological evidence indicating that diets rich in fruits, vegetables, legumes, whole grains, nuts, and other plant foods are associated with a lower likelihood of colorectal neoplasia (26, 27). Unlike studies focusing on individual nutrients or supplements, the PI reflects the overall contribution of phytochemical-rich foods to total dietary energy intake and may better capture synergistic interactions among multiple bioactive compounds (16). The significant dose–response relationship across PI tertiles and the consistency of findings using continuous PI measures strengthen the evidence for an inverse association between phytochemical-rich dietary patterns and CRC. This pattern is mechanistically supported by previous experimental studies showing that phytochemicals such as flavonoids and isothiocyanates activate the nuclear factor erythroid 2-related factor 2 (Nrf2) pathway, enhancing antioxidant enzyme expression and reducing colonic inflammation, which collectively may contribute to suppression of colorectal carcinogenesis (28, 29).

A major strength and distinctive methodological feature of this study is the integration of comprehensive dietary assessment with an extensive panel of objective circulating biomarkers. In addition to evaluating habitual phytochemical intake using a validated FFQ, we simultaneously measured gut permeability biomarkers, inflammatory mediators, oxidative stress indicators, metabolic markers, nutritional parameters, functional outcomes, and tumor-related biomarkers. Furthermore, we explored these pathways using mediation analysis, providing an integrated evaluation of potential biological mechanisms linking dietary phytochemical intake with CRC. The concurrent evaluation of dietary intake and objective biomarkers provides biological support for the dietary assessment and strengthens the interpretation of the diet-biomarker-disease relationships. Few previous studies have simultaneously assessed reported dietary phytochemical intake together with such a broad panel of gut barrier, inflammatory, oxidative stress, metabolic, nutritional, and tumor-related biomarkers in relation to colorectal cancer, making this a methodologically distinctive feature of the current work (30, 31).

Higher PI tertiles were also characterized by lower concentrations of zonulin, LPS, lipopolysaccharide-binding protein, diamine oxidase, intestinal fatty acid-binding protein, and D-lactate. These biomarkers collectively reflect intestinal permeability, epithelial integrity, microbial translocation, and microbial metabolism (32, 33). Diets rich in dietary fiber and phytochemical-containing foods may support microbial diversity and intestinal barrier function (34); however, given the case–control design, these findings should be interpreted as associations rather than evidence of causal biological effects. A proposed mechanism, consistent with previous rodent studies, is that phytochemicals and fermentable fiber may enhance tight junction protein expression (e.g., occludin and claudin-1) and promote butyrate-producing bacteria, thereby potentially reducing gut permeability and endotoxemia-associated colorectal inflammation, although these specific mechanisms were not directly measured in the present study (35).

Similarly, participants with higher PI values exhibited consistently lower hs-CRP, IL-6, TNF-*α*, NLR, PLR, and SII, and these inverse associations remained significant in adjusted regression analyses among patients with CRC. Chronic inflammation is widely recognized as an important component of colorectal carcinogenesis, and experimental evidence suggests that numerous phytochemicals can influence inflammatory signaling pathways (36). The present findings are consistent with an association between phytochemical-rich dietary patterns and a more favorable inflammatory profile. Previous *in vitro* and animal studies have shown that flavonoids and polyphenols suppress NF-κB activation and downstream pro-inflammatory cytokine production while upregulating anti-inflammatory mediators such as IL-10, suggesting a potential mechanistic basis for the observed inverse associations between PI and systemic inflammatory biomarkers, although these intracellular signaling pathways were not directly assessed in the present study (37). Because biomarker measurements were obtained after CRC diagnosis, reverse causation cannot be excluded and biomarker differences may partly reflect disease status rather than pre-diagnostic dietary effects.

Oxidative stress biomarkers demonstrated comparable patterns. Higher PI tertiles were associated with lower concentrations of malondialdehyde and 8-hydroxy-2′-deoxyguanosine and with higher total antioxidant capacity, superoxide dismutase, and glutathione peroxidase. It is noteworthy that the magnitude of the inverse association between the PI and MDA was somewhat more modest among CRC cases compared with other oxidative stress markers, such as total antioxidant capacity or superoxide dismutase. This pattern may reflect persistent lipid peroxidation or disease-related oxidative stress that is not fully attenuated by higher dietary phytochemical intake alone, consistent with the multifactorial nature of oxidative damage in established malignancy. These observations highlight the importance of distinguishing statistical significance from the magnitude of biomarker differences and underscore the need for prospective studies to determine whether the modest reductions in MDA observed here translate into clinically meaningful effects. Critically, the mediation analyses presented here cannot establish causal pathways because all biomarkers were measured after CRC diagnosis. The observed indirect effects may simply reflect disease-associated biomarker alterations (reverse causation) rather than genuine mediation of a diet-disease relationship. We therefore emphasize that these analyses are descriptive statistical decompositions of variance, not evidence of biological mechanisms.

Metabolic biomarkers also differed according to PI. Participants with higher PI values exhibited lower TyG index, HOMA-IR, METS-IR, leptin concentrations, and FGF21 levels, together with higher adiponectin concentrations. Because insulin resistance, adipokine dysregulation, and obesity-related metabolic disturbances have all been linked to colorectal carcinogenesis, these findings further support the broad metabolic profile associated with higher phytochemical intake (38).

Higher PI values were additionally associated with more favorable nutritional and functional characteristics, including higher albumin concentrations, prognostic nutritional index, phase angle, and handgrip strength. Regression analyses confirmed independent positive associations between PI, prognostic nutritional index, and handgrip strength among patients with CRC. Since nutritional status and muscle function are clinically relevant prognostic indicators in oncology, these observations highlight another potentially important dimension of phytochemical-rich dietary patterns (16).

Tumor-related biomarkers, including carcinoembryonic antigen, carbohydrate antigen 19–9, alpha-fetoprotein, and cancer antigen 72–4, generally decreased across increasing PI tertiles. These associations may primarily reflect differences in disease burden rather than direct dietary effects. Nevertheless, because these markers may reflect disease severity rather than dietary exposure alone, their cross-sectional associations should be interpreted cautiously. Previous studies have similarly reported inverse correlations between phytochemical intake and serum tumor markers, with proposed mechanisms including phytochemical-induced apoptosis of tumor cells, reduced synthesis of oncofetal antigens, and modulation of glycosylation processes (15, 39, 40), though these remain hypothesis-generating given the study design.

An additional methodological component of the present study is the exploratory mediation analysis. Biomarkers representing gut barrier integrity, systemic inflammation, oxidative stress, metabolic regulation, and adipokines signaling, demonstrated statistically significant indirect effects in the association between PI and CRC. Although mediation analyses performed within an observational case–control design cannot establish causality or temporal sequencing, they provide hypothesis-generating evidence regarding potential biological pathways that may warrant further investigation in prospective and experimental studies.

This study has several strengths, including a relatively large sample of newly diagnosed CRC patients and frequency-matched controls, comprehensive dietary assessment using a validated FFQ, and evaluation of an extensive panel of gut barrier, inflammatory, oxidative stress, metabolic, nutritional, and tumor-related biomarkers. Exploratory mediation analyses also provided insight into potential pathways. However, several limitations should be acknowledged. The case–control design precludes establishing causality and is susceptible to recall and selection bias. Dietary intake was self-reported and may be affected by measurement error, and dietary habits among cases may have changed due to pre-diagnostic symptoms, introducing reverse causation. Residual confounding cannot be excluded. Importantly, because dietary intake and biomarkers were assessed simultaneously after diagnosis, the observed ‘mediating’ biomarkers likely reflect disease consequences (reverse causation) rather than true causal mediators. Thus, mediation results should be interpreted strictly as exploratory statistical associations, not as evidence of causal pathways. The inverse associations between PI and tumor markers may be confounded by disease severity. With 30 biomarkers examined, some significant associations may be false-positive due to chance. While we used biologically plausible, *a priori* selected biomarkers and examined dose–response relationships, Type I errors cannot be excluded. Thus, all biomarker findings are hypothesis-generating and require confirmation in prospective cohorts, particularly where effect sizes are modest or associations were inconsistent across sensitivity analyses. Prospective cohort studies and randomized dietary intervention trials are warranted to confirm these findings.

## Conclusion

In this hospital-based case–control study, higher dietary PI values were associated with lower odds of colorectal cancer and with more favorable profiles of gut barrier biomarkers, inflammatory markers, oxidative stress indicators, and metabolic biomarkers. The integration of comprehensive dietary assessment with a broad panel of objective biomarkers constitutes a methodological strength of this study. These findings suggest an inverse association between phytochemical-rich dietary patterns and CRC. Prospective cohort studies and randomized dietary interventions are required to confirm these observations and to further investigate the biological pathways underlying the associations reported here.

## Data Availability

The original contributions presented in the study are included in the article/Supplementary material, further inquiries can be directed to the corresponding authors.

## References

[1] Marcellinaro R Spoletini D Grieco M Avella P Cappuccio M Troiano R . Colorectal cancer: current updates and future perspectives. J Clin Med. (2023) 13:40. doi: 10.3390/jcm13010040, 38202047 PMC10780254

[2] Xi Y Xu P. Global colorectal cancer burden in 2020 and projections to 2040. Transl Oncol. (2021) 14:101174. doi: 10.1016/j.tranon.2021.101174, 34243011 PMC8273208

[3] Matsuda T Fujimoto A Igarashi Y. Colorectal cancer: epidemiology, risk factors, and public health strategies. Digestion. (2025) 106:91–9. doi: 10.1159/000543921, 39938491

[4] Ahmed FE. Effect of diet, life style, and other environmental/chemopreventive factors on colorectal cancer development, and assessment of the risks. J Environ Sci Health C. (2004) 22:91–148. doi: 10.1081/LESC-200038263, 16291519

[5] Xie L Li Y Song Z Huang Y Yao Q. Plant-based diet and colorectal Cancer: a systematic review and Meta-analysis of prospective cohort studies. J Gastrointest Surg. (2025) 29:102232. doi: 10.1016/j.gasst.2025.102232, 41015150

[6] Wu B Zhou R-L Ou Q-J Chen Y-M Fang Y-J Zhang C-X. Association of plant-based dietary patterns with the risk of colorectal cancer: a large-scale case–control study. Food Funct. (2022) 13:10790–801. doi: 10.1039/D2FO01745H, 36193696

[7] Shivappa N Godos J Hébert JR Wirth MD Piuri G Speciani AF . Dietary inflammatory index and colorectal cancer risk—a meta-analysis. Nutrients. (2017) 9:1043. doi: 10.3390/nu09091043, 28930191 PMC5622803

[8] Maheshwari S Kumar V Bhadauria G Mishra A. Immunomodulatory potential of phytochemicals and other bioactive compounds of fruits: a review. Food Front. (2022) 3:221–38. doi: 10.1002/fft.129

[9] Gupta C Prakash D. "Phytochemicals as the source of natural immunomodulator and their role in cancer chemoprevention". In: Kesharwani RK Keservani RK Sharma AK, editors. Immunomodulators and Human Health Singapore: Springer (2022) 209–41. doi: 10.1007/978-981-16-6379-6_7

[10] Li Q Geng S Luo H Wang W Mo Y-Q Luo Q . Signaling pathways involved in colorectal cancer: pathogenesis and targeted therapy. Signal Transduct Target Ther. (2024) 9:266. doi: 10.1038/s41392-024-01953-7, 39370455 PMC11456611

[11] McCarty MF. Proposal for a dietary “phytochemical index”. Med Hypotheses. (2004) 63:813–7. doi: 10.1016/j.mehy.2002.11.004, 15488652

[12] Jalalzadeh M Nasli-Esfahani E Montazer M Geravand F Hajian PN Heidari-Seyedmahalle M . The relationship between dietary phytochemical index and novel anthropometric indices and the severity of diabetic foot ulcers in adults with type 2 diabetes: a cross-sectional study. BMC Endocr Disord. (2024) 24:226. doi: 10.1186/s12902-024-01734-x, 39456009 PMC11504297

[13] Ahmadirad H Omrani M Azmi N Saeidian AH Jahromi MK Mirtavoos-Mahyari H . Dietary phytochemical index and the risk of cancer: a systematic review and meta-analysis. PLoS One. (2025) 20:e0319591. doi: 10.1371/journal.pone.0319591, 40173150 PMC11964270

[14] Vincent HK Bourguignon CM Taylor AG. Relationship of the dietary phytochemical index to weight gain, oxidative stress and inflammation in overweight young adults. J Hum Nutr Diet. (2010) 23:20–9. doi: 10.1111/j.1365-277X.2009.00987.x, 19735350 PMC3641567

[15] Bentyaghoob S Dehghani F Alimohammadi A Shateri Z Kahrizsangi MA Nejad ET . Oxidative balance score and dietary phytochemical index can reduce the risk of colorectal cancer in Iranian population. BMC Gastroenterol. (2023) 23:183. doi: 10.1186/s12876-023-02826-z, 37231404 PMC10210348

[16] Hossain MS Wazed MA Asha S Amin MR Shimul IM. Dietary phytochemicals in health and disease: mechanisms, clinical evidence, and applications—a comprehensive review. Food Sci Nutr. (2025) 13:e70101. doi: 10.1002/fsn3.70101, 40115248 PMC11922683

[17] Genua F Raghunathan V Jenab M Gallagher WM Hughes DJ. The role of gut barrier dysfunction and microbiome dysbiosis in colorectal cancer development. Front Oncol. (2021) 11:626349. doi: 10.3389/fonc.2021.626349, 33937029 PMC8082020

[18] Bardelčíková A Šoltys J Mojžiš J. Oxidative stress, inflammation and colorectal cancer: an overview. Antioxidants. (2023) 12:901. doi: 10.3390/antiox12040901, 37107276 PMC10135609

[19] Zhu Y Lu Z Quan J Wu L. Higher dietary phytochemical index is associated with lower disease activity, inflammation, and gut Dysbiosis in patients with ulcerative colitis. Food Sci Nutr. (2025) 13:e71134. doi: 10.1002/fsn3.71134, 41179357 PMC12575179

[20] Fu Y Wang W You J Cheng Z Sun Y Liu Y . Associations of dietary antioxidant and phytochemical indices with cognitive function: mediating roles of basal metabolic rate and systemic inflammation. Antioxidants. (2026) 15:573. doi: 10.3390/antiox1505073, 42193195 PMC13203818

[21] Harmon BE Wirth MD Boushey CJ Wilkens LR Draluck E Shivappa N . The dietary inflammatory index is associated with colorectal cancer risk in the multiethnic cohort. J Nutr. (2017) 147:430–8. doi: 10.3945/jn.116.242529, 28179489 PMC5320401

[22] Cui Q Xia Y Liu Y Sun Y Ye K Li W . Validity and reproducibility of a FFQ for assessing dietary intake among residents of Northeast China: northeast cohort study of China. Br J Nutr. (2023) 129:1252–65. doi: 10.1017/S0007114522002318, 35912695

[23] Ji Y Zhao H. Higher dietary phytochemical index is associated with lower odds of infertility: a case–control study. Front Nutr. (2026) 13:1865952. doi: 10.3389/fnut.2026.1865952, 42500254 PMC13395712

[24] Zheng L Shen Y. Dietary phytochemical index is associated with systemic inflammation, insulin resistance, and gut microbiota-derived metabolites in individuals with obesity: a cross-sectional study. PLoS One. (2026) 21:e0347754. doi: 10.1371/journal.pone.0347754, 42044114 PMC13119903

[25] VanderWeele TJ Tchetgen Tchetgen EJ. Mediation analysis with matched case-control study designs. Am J Epidemiol. (2016) 183:869–70. doi: 10.1093/aje/kww038, 27076669 PMC4851996

[26] Han Y Zhou P Wang L Tang Y Ding Y Yang Y . Linear inverse association between prognostic nutritional index and colorectal cancer risk based on NHANES data. Sci Rep. (2025) 15:25647. doi: 10.1038/s41598-025-10574-1, 40664778 PMC12264100

[27] Zhang X Wu Z Wang X Xin B Hu P Yin Y . Causal associations between dietary factors and colorectal cancer risk: a Mendelian randomization study. Front Nutr. (2024) 11:1388732. doi: 10.3389/fnut.2024.1388732, 38751740 PMC11094808

[28] Clifford T Acton JP Cocksedge SP Davies KAB Bailey SJ. The effect of dietary phytochemicals on nuclear factor erythroid 2-related factor 2 (Nrf2) activation: a systematic review of human intervention trials. Mol Biol Rep. (2021) 48:1745–61. doi: 10.1007/s11033-020-06041-x, 33515348 PMC7925463

[29] Prasad S Kumar V Singh C Singh A. Crosstalk between phytochemicals and inflammatory signaling pathways. Inflammopharmacology. (2023) 31:1117–47. doi: 10.1007/s10787-023-01206-2, 37022574

[30] Ruangwannasak S Khamsai S Pakdeechote P Maneesai P Prachaney P Mothong W . Differential oxidative stress profiles in circulating and Peritumoral adipose tissue across stages of colorectal Cancer. Int J Mol Sci. (2026) 27:707. doi: 10.3390/ijms270707, 41596358 PMC12841512

[31] Verma R Bisen PS Bulló M. Integrated review of cardiometabolic biomarkers and dietary nutrients. J Food Bioact. (2024) 27:44–57. doi: 10.26599/JFB.2024.95027386, 42514360

[32] Perez-Diaz-del-Campo N Castelnuovo G Ribaldone DG Caviglia GP. Fecal and circulating biomarkers for the non-invasive assessment of intestinal permeability. Diagnostics. (2023) 13:1976. doi: 10.3390/diagnostics13111976, 37296827 PMC10253128

[33] Wang S Tian T Zhao H Zhou M Zhang L Gao X . Changes in fecal bacterial and fungal diversity, blood short chain fatty acids, and metabolomics in stage IV colorectal cancer patients. Medicine. (2025) 104:e44102. doi: 10.1097/MD.0000000000004102, 40922299 PMC12419323

[34] Phan MAT Paterson J Bucknall M Arcot J. Interactions between phytochemicals from fruits and vegetables: effects on bioactivities and bioavailability. Crit Rev Food Sci Nutr. (2018) 58:1310–29. doi: 10.1080/10408398.2016.1254595, 27880063

[35] Ganesan K Jayachandran M Xu B. Diet-derived phytochemicals targeting colon cancer stem cells and microbiota in colorectal cancer. Int J Mol Sci. (2020) 21:3976. doi: 10.3390/ijms21113976, 32492917 PMC7312951

[36] Ahmed MB Islam SU Alghamdi AA Kamran M Ahsan H Lee YS. Phytochemicals as chemo-preventive agents and signaling molecule modulators: current role in cancer therapeutics and inflammation. Int J Mol Sci. (2022) 23:15765. doi: 10.3390/ijms232415765, 36555406 PMC9779495

[37] Yahfoufi N Alsadi N Jambi M Matar C. The immunomodulatory and anti-inflammatory role of polyphenols. Nutrients. (2018) 10:1618. doi: 10.3390/nu10111618, 30400131 PMC6266803

[38] Rani D Chatterjee S Goswami PK. An overview on phytotherapeutics for metabolic syndrome: a journey from traditional knowledge to modern clinical validation. J Diabetes Metab Disord. (2026) 25:104. doi: 10.1007/s40200-026-01913-8, 41799535 PMC12965965

[39] Ghoreishy SM Aminianfar A Benisi-Kohansal S Azadbakht L Esmaillzadeh A. Association between dietary phytochemical index and breast cancer: a case–control study. Breast Cancer. (2021) 28:1283–91. doi: 10.1007/s12282-021-01265-6, 34120329

[40] Yin S-Y Yang N-S Lin T-J. Phytochemicals approach for developing cancer immunotherapeutics. Front Pharmacol. (2017) 8:386. doi: 10.3389/fphar.2017.00386, 28674499 PMC5474465

