# Supplementary material for: Dietary phytochemical index and biomarker profiles are associated with colorectal cancer odds: integrating dietary intake with blood biomarkers
Source: Front Nutr. 2026 Sep 15;13:1907770. doi: 10.3389/fnut.2026.1907770 (PMC13619466; doi:10.3389/fnut.2026.1907770)
Supplement: Supplementary file 1 [file Table_1.docx]

**Supplementary Table 1: Linear Regression Analysis of Association Between Phytochemical Index and Inflammatory and Clinical Markers Among Controls (n = 490)**

| **Variable** | **Model 1a: B (95% CI)** | **P-value** | **Model 2b: B (95% CI)** | **P-value** | **Model 3c: B (95% CI)** | **P-value** |
| --- | --- | --- | --- | --- | --- | --- |
| **hs-CRP (mg/L)** | -0.040 (-0.051, -0.028) | <0.001 | -0.037 (-0.049, -0.025) | <0.001 | -0.037 (-0.049, -0.025) | <0.001 |
| **IL-6 (pg/mL)** | -0.046 (-0.061, -0.030) | <0.001 | -0.049 (-0.065, -0.032) | <0.001 | -0.049 (-0.065, -0.032) | <0.001 |
| **TNF-α (pg/mL)** | -0.099 (-0.133, -0.064) | <0.001 | -0.091 (-0.127, -0.055) | <0.001 | -0.091 (-0.127, -0.055) | <0.001 |
| **Zonulin (ng/mL)** | -0.429 (-0.595, -0.263) | <0.001 | -0.369 (-0.542, -0.197) | <0.001 | -0.366 (-0.539, -0.193) | <0.001 |
| **NLR** | -0.033 (-0.047, -0.019) | <0.001 | -0.034 (-0.049, -0.019) | <0.001 | -0.034 (-0.049, -0.019) | <0.001 |
| **PLR** | -2.185 (-3.128, -1.242) | <0.001 | -2.425 (-3.414, -1.436) | <0.001 | -2.417 (-3.406, -1.428) | <0.001 |
| **MDA (µmol/L)** | -0.038 (-0.052, -0.024) | <0.001 | -0.039 (-0.054, -0.024) | <0.001 | -0.039 (-0.054, -0.024) | <0.001 |
| **TAC (mmol/L)** | 0.016 (0.010, 0.023) | <0.001 | 0.016 (0.009, 0.022) | <0.001 | 0.015 (0.009, 0.022) | <0.001 |
| **PNI** | 0.244 (0.125, 0.363) | <0.001 | 0.255 (0.131, 0.381) | <0.001 | 0.256 (0.131, 0.381) | <0.001 |
| **Handgrip Strength (kg)** | 0.189 (0.068, 0.309) | 0.002 | 0.196 (0.070, 0.323) | 0.002 | 0.194 (0.067, 0.320) | 0.003 |

**†** Analysis restricted to controls (n = 490). Regression coefficients represent the expected change in the outcome variable associated with a **1-unit increase** in the Phytochemical Index. Values are presented as **B (95% confidence interval)**.

**a Model 1:** Unadjusted.

**b Model 2:** Adjusted for age, sex, and body mass index (BMI).

**c Model 3:** Adjusted for age, sex, BMI, and total energy intake (kcal/day).
